# Role for Circadian Clock Genes in Seasonal Timing: Testing the Bünning Hypothesis

**DOI:** 10.1371/journal.pgen.1004603

**Published:** 2014-09-04

**Authors:** Mirko Pegoraro, Joao S. Gesto, Charalambos P. Kyriacou, Eran Tauber

**Affiliations:** Dept. of Genetics, University of Leicester, Leicester, United Kingdom; University of Massachusetts Medical School, United States of America

## Abstract

A major question in chronobiology focuses around the “Bünning hypothesis” which implicates the circadian clock in photoperiodic (day-length) measurement and is supported in some systems (e.g. plants) but disputed in others. Here, we used the seasonally-regulated thermotolerance of *Drosophila melanogaster* to test the role of various clock genes in day-length measurement. In *Drosophila*, freezing temperatures induce reversible chill coma, a narcosis-like state. We have corroborated previous observations that wild-type flies developing under short photoperiods (winter-like) exhibit significantly shorter chill-coma recovery times (CCRt) than flies that were raised under long (summer-like) photoperiods. Here, we show that arrhythmic mutant strains, *per^01^*, *tim^01^* and *Clk^Jrk^*, as well as variants that speed up or slow down the circadian period, disrupt the photoperiodic component of CCRt. Our results support an underlying circadian function mediating seasonal daylength measurement and indicate that clock genes are tightly involved in photo- and thermo-periodic measurements.

## Author Summary

The circadian clock consists of an extensive genetic network that drives daily rhythms of physiological, biochemical and behavioural processes. The network is evolutionary conserved and has been extensively studied in a broad range of organisms. Another genetic network constitutes the photoperiodic clock and monitors the seasonal change in day-length. Here, we address a major and long-standing question in chronobiology: whether the circadian clock is involved in photoperiodic timing, also known as the Bünning hypothesis. *Drosophila*, as with many other insects in temperate regions, exhibits a photoperiodic response that allows the insect to anticipate and survive the winter. Here we show that the cold-tolerance of the fly is regulated by the photoperiod. We use this phenotype to test day-length timing in various circadian clock mutants and observe that in null clock mutants, the photoperiodic response is abolished, whereas in mutants that exhibit short or long daily cycles, the photoperiodic response is modified, further supporting a circadian-clock function. Overall, these results provide the first evidence in *Drosophila* that support for the Bünning hypothesis, and pave the way for the genetic dissection of seasonal timing in *Drosophila melanogaster*.

## Introduction

Seasonal changes in day-length provide a reliable environmental cue used by many temperate species to adapt to their fluctuating environments. While the available evidence suggests that changes in day-length are monitored by an internal photoperiodic timer [1], intensive studies of photoperiodicity in animals over the last 80 years have yet to identify an underlying molecular mechanisms [2] (although significant progress has been made in plants and mammals [3]–[5]).This is in marked contrast to the level of understanding of the circadian timer that regulates daily rhythms, where studies in various model organisms, particularly *Drosophila*, led to the discovery of principles and molecules that are highly conserved in diverse phyla [6].

The Bünning hypothesis [7] invoked a link between the circadian and the photoperiodic mechanisms and suggested that circadian rhythmicity is required for day-length measurement. Bünning's original model assumed that circadian oscillations consist of light (‘photophil’) and dark (‘scotophil’)-requiring phases. In short days, ambient light is present only during the photophil phase, and the dark phase is not exposed to light. As days become longer, light coincides with the scotophil phase. The relative size of the photophil and scotophil phases encodes the critical photoperiod (time of the year) that induces the seasonal response. A modified version of this model was later named the ‘external coincidence model’ [8]. An alternative hypothesis, the ‘internal coincidence model’, was also proposed, where light plays only an indirect role, and the critical photoperiod is encoded by unique phase relationships between two internal oscillators. Several experimental protocols have been devised to test the Bünning hypothesis, one of which is the Nanda-Hamner protocol [9], which employs exotic light-dark cycles of ultra-long periods (T>72 hr). If the seasonal response peaks at 24 hr intervals (‘positive Nanda-Hamner’), a link, not necessarily causal, with the circadian system in photoperiodic timing is indicated [9], [10].

*Drosophila melanogaster*, which was instrumental in identifying higher eukaryotic circadian clock genes [11], also exhibits a photoperiodic response [12], providing an opportunity to test the link between the two timers. This response is manifested as a developmental arrest of the ovaries (i.e. reproductive diapause) under short (autumnal) days and lower temperatures, presumably enhancing the fly's ability to survive the winter in temperate regions. Although Nanda-Hamner experiments in *Drosophila* revealed an underlying 24 h oscillation [13], experiments using the *period (per)* clock mutants [12] suggested that the two systems are independent, as the *per* null mutants were still capable of discriminating between long and short days (albeit with a shifted critical day-length). Later, natural allelic variation in the *timeless* (*tim*) locus was associated with diapause in *Drosophila* [14], [15], and in *Chymomyza costata* [16]. Knockdown of *per* and the positive circadian regulator, *cycle*, in the bean bug *Riptortus pedestris* by RNAi caused simultaneous disruption of both circadian output (cuticle deposition rhythm) and photoperiodic diapause [17]. In *Drosophila triauraria*, genetic variation in *tim* and *cry* (but not in *per*, *Clk* or *cyc*) was significantly associated with the photoperiodic response [18]. Yet, because the impact of a given clock gene mutation on the photoperiodic response can be interpreted as a pleiotropic effect on diapause, the application of the Bünning hypothesis to these results has been questioned [19].

Given the shallow photoperiodic diapause of *D. melanogaster* [14], we have sought an alternative seasonal phenotype in this species that could be used for testing Bünning's hypothesis. Measuring chill comma recovery times (CCRt) is an established approach for studying insect thermal adaptation [20]. Here, we build on the earlier observation that flies raised in different photoperiods show differing CCRt [21], and use this phenotype to test day-length timing in various circadian clock mutants.

## Results

**Figure 1:**
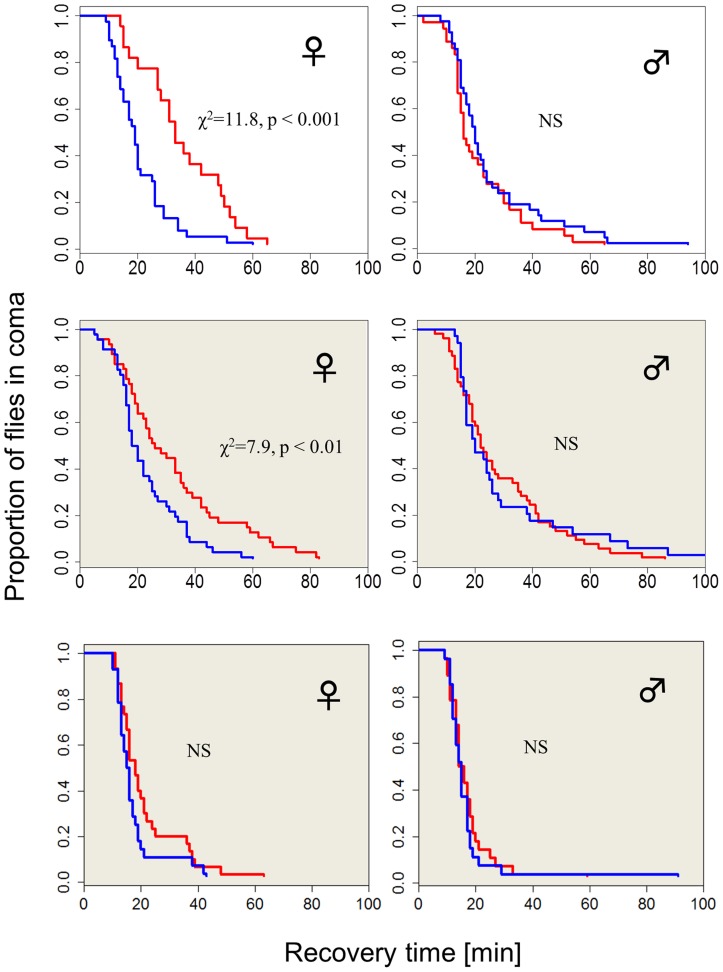
Photo/thermoperiodic-dependent temperature-tolerance in *Drosophila*. Survival curves showing recovery from coma of wild-type flies (Hu) that were developed in either short winter-like (blue) or long, summer-like (red) daylengths (N_1_, N_2_ = 38, 22). Flies exposed to diurnal light and temperature cycles are shown in the top panel, flies exposed only to the thermoperiod are depicted in the middle panel (shaded grey, N_1_, N_2_ = 47, 46). Wild-type females (but not males) that develop under short days exhibit significantly faster chill-coma recovery times (CCRt) than flies that were raised under long day photo- or thermoperiods. In a control experiment (bottom panel), where the flies are maintained in the photoperiod but in covered vials (DD), and with the thermoperiod overridden by a reversed temperature cycle (i.e. Δ T = 0°C), there is no detectable difference in CCRt (N_1_, N_2_ = 29, 24).

The chill-coma recovery times (CCRt) of wild-type flies raised at different photoperiods is sexually dimorphic (Figure 1). Females that developed under short winter-like photoperiod exhibit significantly shorter CCRt than females that were raised under long summer-like photoperiods (log rank test, χ^2^ = 11.8, df = 1, p<0.001). However, CCRt did not differ significantly between males raised on long vs. short days (Figure 1). Females kept in covered vials (in darkness, DD) within the same chambers also showed a moderate but significant differential response, driven by the low-amplitude (∼2°C) thermoperiod that was generated by the lighting system (log-rank test χ^2^ = 7.9, p<0.01, see Methods). In another set of experiments where the heat cycles produced by the two photoperiods were offset by a counteracting temperature cycle, there was no significant difference in the CCRt between long and short photoperiods in females kept in covered vials (in darkness, DD). The difference in median CCRt between long and short days in females exposed to light (driven by both photoperiod and thermoperiod) was twice as large as the difference exhibited by the thermoperiod only (DD) females (14 vs. 7 min). This difference in photoperiod was significant after statistically accounting for the temperature effect (via non-parametric ANCOVA, W = 664, p<0.001) confirming the interaction between photoperiod and thermoperiod.

We also tested the CCRt of wild-type females over a range of five photoperiods (Figure S1). For short photoperiods 8, 10 and 12 hr, the median CCRt was 15 min (with 95% CI overlapping 13–33). In the 14 hr photoperiod, the median was 13 min (12–19) and at 16 hr was 24 (20–30). Thus, an intermediate photoperiod (comparable to the critical day length in diapause studies) would lie within the 14–16 hr interval. We also examined the association between the CCRt and diapause propensity (Figure S2). Newly emerging females were maintained in diapausing inducing conditions (Methods). After 12 days their CCRt was tested, followed by ovary dissection for determining their reproductive state. The CCRt did not differ significantly between diapausing (n = 82) and non-diapausing (n = 39) females (χ^2^ = 0.1, df = 1, p = 0.70).

There was however a significant difference in female weight between long vs. short day (Figure S3): Fresh weight in short days was higher (F_1,8_ = 9.68, p<0.05), due to higher water content (F_1,8_ = 6.57, p<0.05), since dry weight was similar (F_1,8_ = 4.13, p = 0.07). Males also showed weight difference between long and short day, which was significant, both for fresh and dry weight (Figure S3). Thus, size or water differences cannot fully explain the photoperiodic CCRt response (which is absent in males). In addition, the CCRt of males resembles that of females in short days (although the size difference between the sexes is substantial).

**Figure 2:**
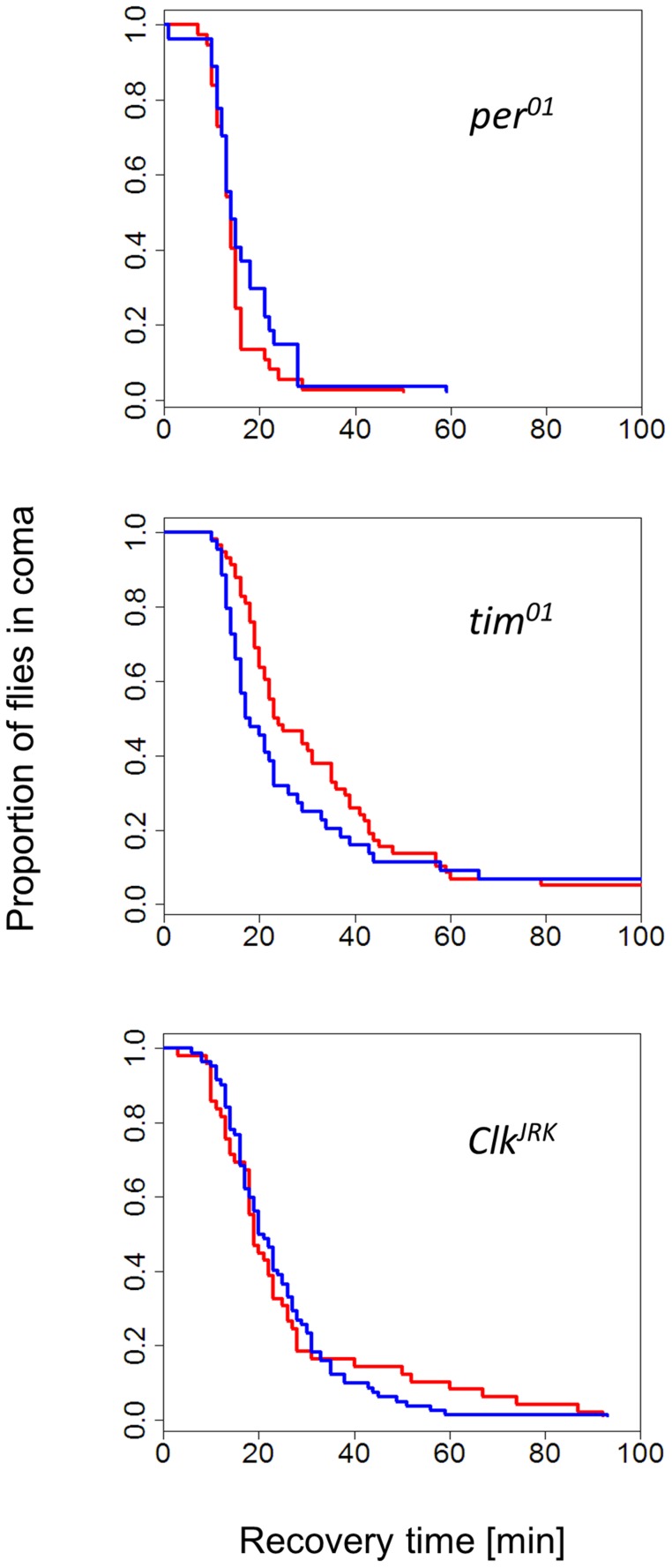
Photo/thermo-periodic response is disrupted in clock mutants. In clock mutant females *per^01^* (N_1_, N_2_ = 37, 27), *tim^01^* (N_1_, N_2_ = 58, 44), *Clk^Jrk^* (N_1_, N_2_ = 49, 82) the differential CCRt response to day-length (flies exposed to photo/thermo-period cycles) is abolished. All strains have the same genetic background (Hu). Flies were developed in either short winter-like (blue) or long, summer-like (red) day-lengths.

The finding that female flies were able to discriminate between long and short days provided us with the opportunity to test the role of circadian clock genes in this response. The CCRt of females from congenic strains carrying *per^01^*, *tim^01^*, and *Clk^Jrk^* mutations did not show any photo/thermoperiodic effect (Figure 2). In general, the mutant curves resembled the WT short-day response (median CCRt 19 min, range 15–25). For example, in long days, the median recovery time for *per^01^* was 14 min (13–15), for *Clk^Jrk^* 19 min (18–23) and for *tim^01^* 23.5 min (21–35). We did notice however, some differences in cold-tolerance among the mutants, particularly between *per* and the two other mutants (note the overlapping CIs).

To gain insight into the metabolic correlates of the photoperiodic response, we measured glycogen, free fatty acids and protein levels (Figure S4). In wild-type females, glycogen was significantly higher in long days (F_1,46_ = 4.05, p = 0.05), while four time points taken during the day did not show any significant differences (F_1,46_ = 2.22, p = 0.14). In contrast, in *Clk^Jrk^* females glycogen levels did not differ between photoperiods (F_1,22_ = 0.85, p = 0.37). For free fatty acids, neither the photoperiod nor the time of the day showed significant differences (Figure S4) in WT or *Clk^Jrk^*. Similarly, total protein also did not differ between photoperiods, but intriguingly there was a significant photoperiod:Zt interaction in the *Clk^Jrk^* mutants (F_2,21_ = 7.41, p<0.01).

**Figure 3:**
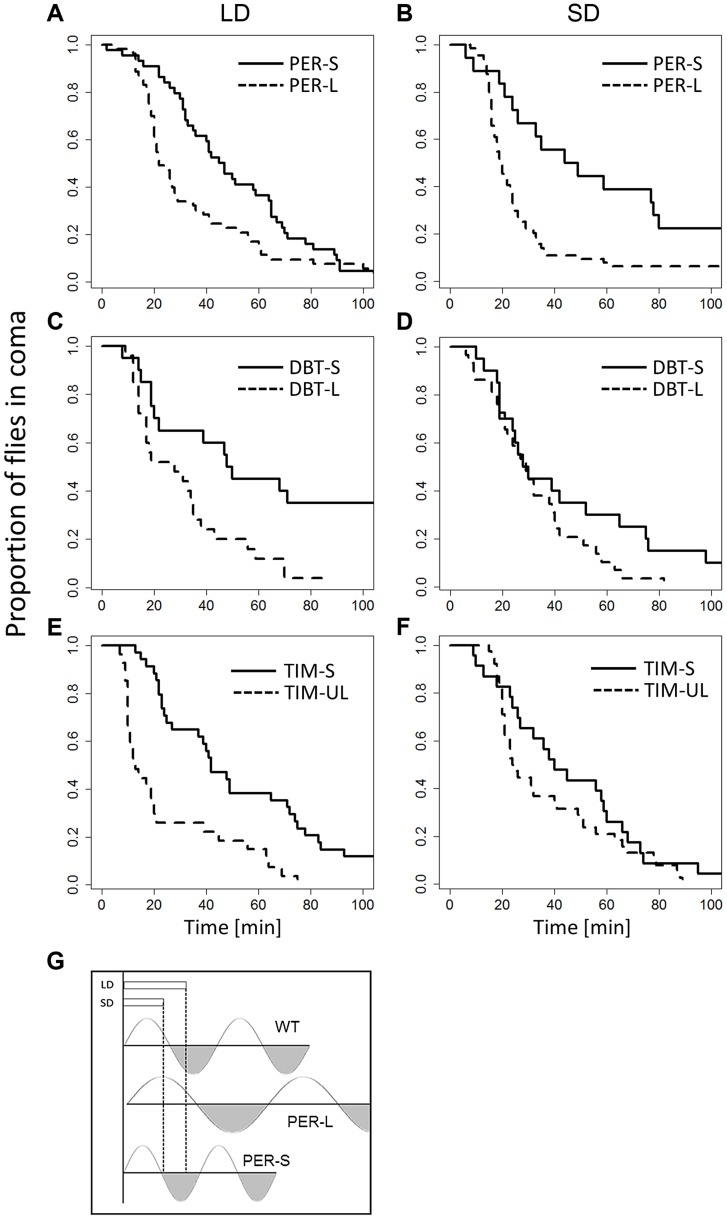
CCRt of long and short period alleles of clock genes. The response of mutant females maintained in long (left column) and short (right column) days (thermoperiods) is depicted. Response of *per^L^* and *per^S^* is shown in (A) (44, ÷2 = 11.9, p<0.001; N_1_, N_2_ = 53) and (B) (χ^2^ = 11.1, df = 1, p<0.001; N_1_, N_2_ = 53,44). The response of *dbt^L^*, *dbt^S^* is depicted in (C) (χ^2^ = 9.5,df = 1, p<0.05, N_1_, N_2_ = 29, 20) and (D) (χ^2^ = 2.8, df = 1, p = 0.093; N_1_, N_2_ = 29,20). The response of *tim^S1^* and *tim^UL^* is shown in (E) (χ^2^ = 18.4, df = 1, p<0.001, N_1_, N_2_ = 27, 51) and (F) χ^2^ = 1.7, df = 1, P = 0.19; N_1_, N_2_ = 38, 23). G. Schematic diagram showing Bünnings' external coincidence detector in wild-type (WT) and mutant flies. In WT short days coincide with the photophil phase while long day extend to the schotophil phase (note that daylength may be either encoded by photoperiod or the thermoperiod). In long-period mutants, long days still coincide with the photophil phase, and are interpreted as short days, leading to a constitutively short day response. In short period mutants the photophil phase may be brief so even short day are interpreted as a long one. In both type of mutants, long and short daylength may coincide with the same phase of the detector, resulting in loss of the photo/thermo-periodic response.

The availability of mutants that exhibit a long or short circadian period provided us with a further opportunity for testing the Bünning hypothesis. We compared long and short mutant alleles of three genes (Figure 3): *per^L^*, *per^S^* (28.8 vs 19.3 hr circadian period [22]), *dbt^L^*, *dbt^S^* (26.8 vs 19.3 hr, [23]) and *tim^UL^*, *tim^S1^* (32.7 vs 21.1 hr, [24]). An omnibus ANOVA for analysing the data of all genes simultaneously including experiments at different photo/thermo-periods resulted in a highly significant ‘allele’ factor (short v long period, F_1,813_ = 28.46, p<0.001) but not ‘photoperiod’ (F_1,813_ = 0.57, p = 0.45). There was no significant gene:photoperiod interaction (F_2,813_ = 1.39, p = 0.25). In all three genes, the CCRt of the long alleles was consistently shorter (Figure 3, particularly evident in *per* mutants), in both photoperiods, suggesting that the long period mutant perceive the day as shorter compared to short period mutants. This result is consistent with Bünning's original model (Figure 3). We also observed that CCRt does not fluctuate significantly throughout the day in WT, (χ^2^ = 1.4, df = 3, p = 0.7; Figure S5) so that the differences between the mutants is not due to our sampling of CCRt at different subjective phases. We have also compared the average phase angle of the light-entrained activity of the long and the short-period mutants (Figure S6). We estimated the phase values using the pooled locomotor activity profile (16–35 flies in each experiment), averaged over four days. Across genes (n = 3, each tested for two alleles, at two photoperiods, giving 12 data points), there was a significant difference between the long and the short alleles for both morning peak (MP; F_1,9_ = 140.8, p<0.0001) and evening peak (EP; F_1,9_ = 18.3, p<0.01). Both the MP and EP were advanced in short allele flies, but for the MP, the advance was enhanced in short day, resulting in significant allele: photoperiod interaction (F_1,9_ = 105, p<0.001).

**Figure 4:**
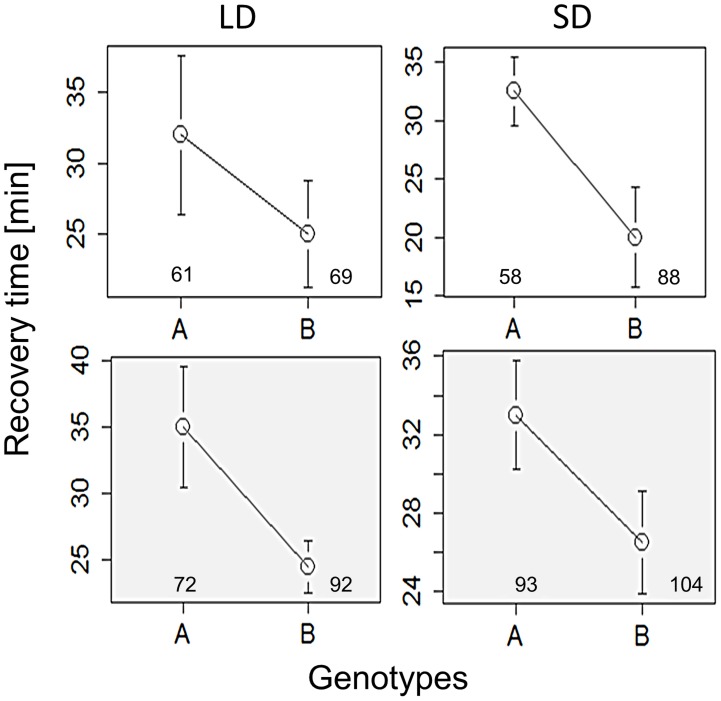
Chill coma recovery in *per* splicing transgenic flies. Medians of CCRt under long (left panels) and short day (right panels) driving by photo/thermo-periods, or by the thermoperiod only (shaded grey). Data were pooled for each of the two strains expressing each of the splice variant (A, B). Flies carrying Type B′ transgene, which is locked into the constitutive *per* 3′ UTR splice mode, show consistently shorter CCRt. Error bars represent SE. Number of females is also shown.

We have also explored the role of alternative splicing in the *per* locus that was previously associated with seasonal adaptation [25]–[27]. Specifically, under low temperatures, as well as short photoperiods, the splicing level of intron 8 in the 3′UTR of *per* is increased. To test the role of *per* splicing in CCRt, we used transgenic lines in which the splicing signal is missing and the intronic sequence cannot be spliced (type A), or a construct that does not contain the intron (type B′) [28]. The *per^A^* and *per^B′^* transgenes were expressed in *per^01^* flies. For each transgene two independent insertion lines were tested (see Methods) and their data were pooled. As shown in Figure 4, flies carrying the type B′ transgene showed shorter CCRt both under long or short photo/thermoperiod or thermoperiod alone. ANOVA incorporating all the conditions in which the transgenic flies were tested revealed a significant splice type factor (F_1,634_ = 8.53, p<0.01). In contrast to the wild-type Hu strains, the splice variants were not photoperiodic (Figure 4), presumably due to the different genetic background of the transformant flies (*yw*). The control lines *per^G^* did show a thermoperiodic response (in DD; χ^2^ = 10.1, p<0.01; N_1,_N_2_ = 128,112), but were not photoperiodic (LD: χ^2^ = 0.1 p = 0.77; N_1,_N_2_ = 110,121). In general the CCRt of *per^G^* resembled the response of *per^A^* with relatively longer medians (LD:SD = 33,35 min; in DD, LD:SD = 60, 39.5 min).

Taken together, the results indicate some effect on cold tolerance for *per* splicing, further supporting the notion that the circadian clock or signalling to the circadian clock is involved in this seasonal adaptation.

## Discussion

The chill-coma recovery test has been used in various insect species for studying cold tolerance and adaptation [29]. Recent studies in *D. montana* have demonstrated that the CCRt in this species is under photoperiodic regulation [30], [31], consistent with the expectation that the autumnal shortening of the day induces various process, including nutrient regulation and reserve accumulation that allows the flies to survive the winter.

Here, we have shown that a similar day-length regulation is present in *D. melanogaster*, and we exploited this response to study the link between the circadian clock and seasonal timing. While our experiments have not disentangled entirely the thermo- and photo-periodic effects, the difference in CCRt response in LD (day-length encoded by both photoperiod and thermoperiod), and DD (thermal information only) would suggest that both cues contribute to the response (Figure 1).

We show that in clock mutant strains *per^01^*, *tim^01^* and *Clk^Jrk^* the day-length measurement is disrupted. The lack of photoperiodic CCRt in *per^01^* is in apparent contradiction to the previously reported photoperiodic diapause in this mutant [12]. Differences between the CCR and diapause phenotypes may represent two separate photoperiodic circuits that use different genetic networks, a situation which resembles the different circadian locomotor and eclosion circuits [32], [33]. Interestingly, Helfrich-Förster [34] analysed the bimodal locomotor activity profile of *per* mutants and suggested that the morning peak is derived from a *per*-independent circadian component (see also [35]), and that this component might be involved in photoperiodic timing. In addition, a recent study [36] using temperature entrainment suggests that *per^01^* (and *tim^01^*) are not entirely clockless.

However, it should be noted that the reported photoperiodic response of diapause in *per^01^* (and *per* deficiency flies) is altered because the critical day length (CDL) for inducing diapause is several hours shorter than in wild-type [12], [37]. In our experiments, the *per^01^* mutants mirror their CDL and exhibit the shortest CCRt. This correlation may reflect the situation in the wild, where populations in colder environments (presumably more cold-adapted flies) would be expected to show a shorter CDL that will trigger diapause later in the season.

The substantial difference in CCRt between long- and short circadian period alleles is particularly informative (Figure 3). The constitutively ‘short-day’ response of the long-period mutants fits well with the ‘external co-incidence’ model underlying day-length measurements (Figure 3G). In wild-type flies, short days coincide with the photophil phase of the pacemaker, while long days extend to the scotophil phase of the oscillations. In long-period mutants (where photophil phase is longer), various daylengths always coincide with the photophil phase and are interpreted as short days, while in short-period mutants both long and short day-length may overlap with the scotophil phase and be interpreted as long days. The main weakness of this model is the requirement for a uniform waveform of the oscillation under long and short days. In *Drosophila* however, this model is unlikely to be valid, as the oscillation waveform of overt rhythms (locomotor activity) and level of clock proteins change during the seasons [35], [38]. Furthermore, the model (Figure 3) disregards the entrainment of the mutant oscillation during the seasons [35]. Depending of the phases of the mutants, different outcomes may be predicted (Figure S7). Indeed, we have observed a consistent phase difference between long and short period alleles (phase advance in short-period alleles, Figure S6). Interestingly, our data mirrors an early study of the eclosion rhythm of *D. pseudoobscura* [39], where wild-type flies kept in T cycles longer than the circadian period (resembling the short-period mutants in our study, kept under 24 hr cycle) showed a phase advance, and flies kept in T<τ exhibited phase delays. While the link between the circadian phase and the photoperiodic response is yet not clear, the different photoperiodic phenotypes of the slow and fast clock mutants seems to suggest a causative role for the circadian pacemaker in day-length measurement, but further experiments are required to identify the underlying model (external- vs. internal-coincidence, or any other model). A further analysis of the critical day length of the CCRt (Figure S1) in the long and short clock mutants may provide more insights about the link between the circadian system and the photoperiodic timer, and this will be published elsewhere. Similarly, using the Nanda-Hamner or Bünsow protocols [9] would provide more ways for testing the circadian role in the photoperiodic CCRt.

Our results also show that the regulation of *per* splicing, a process which was previously implicated in the fly's seasonal response [26], [27], is also involved in the CCRt, as flies carrying the type B′ transgene exhibited shorter recovery times compared with flies carrying type A (Figure 4). This fits well with the enhanced splicing at cold conditions in the type B′ transgenic *per* but contrasts with the observation that the seasonal locomotor activity profile of the two type of transgenic flies is similar [25]. This may suggests that retention or removal of the *per* 3′ intron is affecting the CCRt, while the splicing process itself is critical for the cold-induced phase advance in the locomotor activity rhythm. Our results reflect the seasonally adaptive nature of *per* splicing because during the autumnal shortening of the photoperiods (and decreasing temperatures), *per* splicing will inevitably increase [26], [35]. This will lead to locomotor changes but also, we suggest, to further physiological changes that may allow the fly to tolerate lower temperatures, as manifested by shorter CCRt (Figure 4).

The circadian clock and the photoperiodic timer appear to act as two modules, each consisting of a group of functionally related genes [40]. Genes interact primarily with genes within the same module, although individual genes may have an effect on the other module. Such pleiotropic effect of individual clock genes on diapause was proposed as an alternative explanation to the “Bünning hypothesis” [10]. In the current work however, the fact that a battery of circadian clock genes are implicated in daily light and temperature measurements strongly indicates that the two modules functionally interact, not simply as isolated pleiotropic effects of one gene on another module. Under pleiotropy, knockdown of different clock genes may results in different outcomes. For example, RNAi targeting either *per* or *cyc* in the bean bug *Riportus pedestris* led to aberrant photoperiodic response, but in opposite directions [17]. In contrast, in our experiments all null mutants exhibit the same trend (loss of long day response; Figure 2), further suggesting that the Bünning hypothesis provides the most parsimonious explanation.

The shortening of CCRt in flies exposed to short photoperiod, which was also recently reported for *D. montana* [30], reflects an enhanced cold tolerance acquired during development. This cold acclimation is presumably mediated by cold hardening, a process which involves changes in phospholipid fatty acids composition of cell membranes [41], [42], as well as polyols, sugars and other metabolites [43]. The ecological relevance of the improved cold tolerance following cold hardening was previously demonstrated in enhanced *D. melanogaster* survivorship [44] and reproductive behaviour [45] in flies primed for experiencing low temperatures.

Beyond demonstrating the role of the circadian clock in seasonal timing, our results here juxtapose the CCRt phenotype against female reproductive diapause, the classic readout for insect seasonality [46]. While cold hardiness is often associated with diapause our results suggest that the responses are triggered by different mechanisms (Figure S1). Similarly studies in *D. montana* shows that the CCRt is not always correlated with diapause and is strain-dependent [30].

For studying seasonal timing, analysing diapause involves extremely laborious dissection of ovaries, and the binary nature of the phenotype (diapause status) requires the processing of large sample sizes for detecting appreciable effects. In contrast, the automated CCRt phenotyping allows for high-throughput screening and the protocol requires that flies are maintained at 20°C, which is more conducive to GAL4 misexpression studies than diapause experiments that are usually performed at 12°C. CCRt thus provides a powerful and efficient method for dissecting the genetic and anatomical basis of seasonal timing in *D. melanogaster*.

## Methods

### Fly strains

The strains *per^01^*, *tim^01^*, *Clk^Jrk^* were used. All strains were crossed to an isofemale strain originating from a wild Dutch population in Houten [14]. The progeny were screened for individuals carrying the mutation using PCR genotyping as previously described [47] and backcrossed to Hu, a process which was repeated 8 times, resulting in all the mutations inserted in the genomic Hu background (>99%) that also carries the *ls*-*tim* natural allelic variant [14]. Mutants were made homozygous by further crossing to balancer strains (also on a Hu background). In addition, the strains *per^L^* and *per^S^* [13], *dbt^S^*, *dbt^L^* [23], *tim^UL^* and *tim^S1^* [48] were used (the genetic background of these mutants is not Hu). The mutant's circadian locomotor activity was verified at 19.5°C, which was used for the CCRt experiments (Figure S6).

To investigate the effect of *per* splicing on CCRt we used congenic transgenic lines that generate either type A (PERA-18, PERA-29) or type B′ (PERB′-11, PERB′-12) *per* RNA and have been used to rescue *per^01^* flies. We have also tested transgenic flies expressing both type A and type B′ (*per^G^*). These lines have been described previously [28]. All strains were maintained at 25°C in LD 12∶12 on a standard cornmeal media.

### The CCR protocol

Around 100 flies were kept on egg-collection food for 18 hr, and four replicates of 40 eggs each were transferred to new vials. The vials were placed in either long (16 hr) or short (8 hr) day using fluorescent light boxes. Temperature within the light boxes fluctuated during the LD cycle, due to heat produced by the florescent light, from 21°C during the light phase to 19°C during scotophase. Temperature was monitored by data loggers (Tinytag UK). Each experiment was replicated twice with two different light boxes (total of 8 vials). DD samples (vials covered by aluminum foil, providing constant darkness) were also included, and were used for analysing the effect of the thermoperiod (2°C cycling). The flies were developed under these conditions for 20 days, and emerging adults (age 3–4 days) were tested for their chill coma recovery as follows: At ZT 3.5 (ZT, Zeitgeber time, hr after lights on) the flies were anesthetized by ice, sexed and transferred individually to glass activity tubes (outer diameter = 5 mm, 80 mm) and cotton plugs were used to place the fly at the middle of the tube. The flies were kept on ice at 4°C for 3 hours. At ZT 6.5, the glass tubes were loaded into the Drosophix locomotor activity monitor (Padova, Italy), which was previously described [27] at 25°C. This infra-red based system uses the same glass tubes used by the Trikinetics system, but the space of the tube was reduced to 2 cm by cotton plugs (i.e. the fly was approximately 1 cm from the light beam).

The loading time (t_0_) for each fly was recorded by the system. A custom written script in “R” [49] was used to calculate the recovery time (CCRt), by subtracting t_0_ from the time of first movement detected by the system (consequently, our calculated recovery times are slightly longer, by definition, from previous studies, where recovery time was defined as the time in which the fly was first observed standing).

Given the exponential distribution of the CCRt, these data are best analysed by survival curves. We used the *Survival* R library to fit Kaplan-Meier curves, and log-rank tests to compare the different curves, using χ^2^ statistics with one degree of freedom [50] We used ANOVA to test the contribution of various factors across different experiments (e.g. day-length, sex, photo- vs. thermoperiod entrainment, etc.). For this purpose we used log transformation for variance stabilisation. To compare the effect of photoperiod after correcting for temperature effect, a non-parametric ANCOVA was carried using the Quade procedure [51]. Briefly, the CCRt irrespective of group membership (photo- or thermo-periodic) were ranked, and regressed over day-length. The residuals were then compared by the Wilcoxon rank sum test.

### Diapause

Male and female flies (Hu genetic background) were collected within a six hour post eclosion window and placed under 8∶16LD (light∶Dark) at 12.2±0.2°C. After 12 days, CCRt was measured and immediately followed by dissection of the female ovaries in PBS. Reproductive arrest was determined as previously described [14]. The diapause level in females that were maintained under the same conditions but were not tested for CCRt was not significantly different from females that were exposed to coma inducing temperature (F_1,14_ = 0.44, p = 0.51), indicating that the cold treatment did not contribute to diapause state.

### Free fatty acid, glycogen and proteins measurements

Flies developing under LD and SD (see CCR protocol) were collected (4 days old) at four time points (Zt1, 7, 13 and 19) and immediately frozen in liquid nitrogen and stored at −80C. The fresh weight of 10 individuals was recorded after a 5 min thaw in ice with a precision balance (Precisa180A). Glycogen, proteins and free fatty acid content were measured in these samples and expressed as µg or nmol per fresh weight. Glycogen concentration was obtained from samples that were homogenized in 100 µl of water in ice for 30 sec. After centrifugation at top speed for 5 min (4°C), the homogenates (10 µl were saved for protein assay) were boiled for 5 min and Hydrolysis buffer was added to final volume of 120 µl (Sigma-Aldrich, MAK016). Glycogen content was assayed by colorimetric reaction (570 nm) after treatment with Hydrolysis Enzyme and Development Enzyme (Sigma-Aldrich, MAK016). Glucose background was removed from each sample. 10 µl of homogenates were diluted 10 times in water and proteins quantified spectrophotometrically (595 nm) using Bradford reagent (10 µl diluted samples +290 µl reagent; Sigma-Aldrich, B6916). Total proteins were quantified using a BSA (10 mg/ml) standard curve. The free fatty acids were isolated from samples homogenized for 30 sec in ice in 200 ul chloroform-1% Triton X-100. After 10 min centrifugation the organic phase was isolated and vacuum dried for 30 min to remove the chloroform. The lipids were dissolved in 200 ul of fatty acid buffer (ABCAM ab65341). Free fatty acid content was assayed by colorimetric reaction (570 nm) after Acyl-CoA synthesis (ABCAM ab65341). FLUOStar omega plate reader was used for both colorimetric reactions and the Bradford assay.

### Fresh, dry weight and water content

Fresh weight (FW, g) was measure from samples collected at Zt 3.5 (LD and SD) using a precision balance (Precisa180A). Dry weight (DW, g) was measured after desiccating the sample at 60°C for 3 days. The difference between FW and DW indicate the water content (WC, g).

### Locomotor activity

The locomotor activity of 3–4 days old virgins was measure at 19.5±0.5°C using the Trikinetics system. The activity of flies was recorded during 4 days entrainment (either LD or SD) follow by 5 days of constant darkness (DD). The DD activity was also used to calculate the flies' circadian period of activity using “Cosinor analysis” [52], which employs the least squares method to fit a sine wave to a time series. Monte Carlo simulations (n = 100) were used to estimate 99% significant level. For phase analysis, the morning and the evening peak were recorded and converted into degrees. Because the data are circular, large angles (>270°) were converted to negative values (subtracting 360°).

## Supporting Information

Figure S1 The effect of photoperiod on CCRt. Survival curves showing recovery from coma of wild-type females (Hu) that were developed in photoperiods 8–16 hr (n = 19–41). Median CCRt of 8–14 hr photoperiod is similar 13–15 min, while for 16 hr, the median is 24 min (see text).
(TIF)

Figure S2 Comparison of CCRt in diapausing and non-diapausing females. Survival curves showing recovery from coma of wild-type females (Hu) following 12 days in diapause inducing conditions. Following CCRt measurement the females were dissected and the reproductive state was determined. Diapausing (n = 82) and non-diapausing (n = 39) females show similar CCRt.
(TIF)

Figure S3 Fresh/dry weights of Drosophila under different photoperiods. The fresh weight (top), the dry weight (middle) and the water content (bottom) were measured in females (left bars) and males (right bars) raised in long (16 hr) and short (8 hr) day. Measurements are based on 4–5 replicate pools of 10 flies. The error bars represent SE.
(TIF)

Figure S4 The effect of photoperiod on metabolite level. The level of glycogen (top), free fatty acids (middle) and total protein (bottom) was measured in female wild-type (HU) and mutant (*Clk^JRK^*) flies, under long and short day. Samples were collected at four different time points. Glycogen assays are based on 3–6 pools of 10 flies each. Free fatty acids assays are based on 2–3 replicates (10 flies each), and total protein 3–9 pools (10 flies each). Error bars represent SE.
(TIF)

Figure S5 CCRt at different times of the day. Survival curves of wild-type females (Hu) showing similar recovery time from coma at different Zts. Females were developed at LD 12∶12 (n = 44–65 flies).
(TIF)

Figure S6 Locomotory activity profiles of clock mutants. The activity profiles of mutant female flies in long (left) and short (right) day is shown, followed by one day in DD (n = 16–35 flies). Experiment carried at 19.5°C, which was also used in the CCRt experiments.
(TIF)

Figure S7 Predicted behaviour of long and short mutants under external coincidence model. A: Assuming that mutants and WT flies have the same oscillation waveform, that the waveform remains unchanged under long and short photoperiods, the scotophil and photophil phases of each strains may lie at different time of the day (*per^L^* delayed compared to *per^S^*). B: Under long days, *per^L^* mutants have a larger portion of their scotophil phase in darkness compared to *per* ^S^, that leads to a short day response (better cold adapted than the other strains). This fits the hypothesis shown in Figure 3. C: Under short-days, however, *per^S^* and WT flies have the entire scotophil phase in darkness, and *per^S^* mutants have in addition the largest part of the photophil phase during the day. Therefore, *per^S^* mutants should be best winter-adapted. This was not found in the experiments. Consequently, the external coincidence model can only explain part of the results.
(TIF)
