# Supplementary figures and images for: Role for Circadian Clock Genes in Seasonal Timing: Testing the Bünning Hypothesis
Source: PLoS Genet. 2014 Sep 4;10(9):e1004603. doi: 10.1371/journal.pgen.1004603 (PMC4154681; doi:10.1371/journal.pgen.1004603)

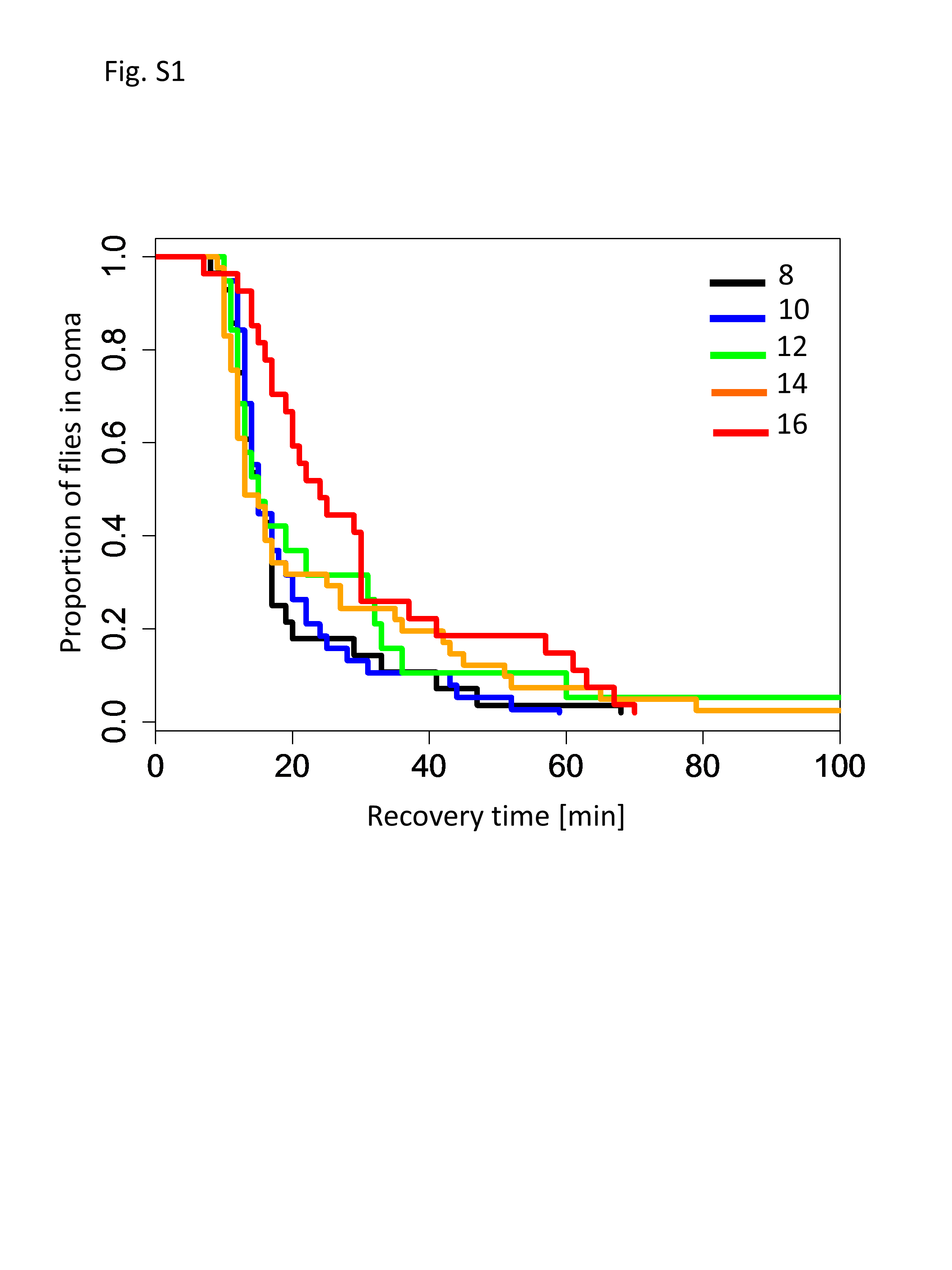

Supplement: Figure S1 — The effect of photoperiod on CCRt. Survival curves showing recovery from coma of wild-type females (Hu) that were developed in photoperiods 8–16 hr (n = 19–41). Median CCRt of 8–14 hr photoperiod is similar 13–15 min, while for 16 hr, the median is 24 min (see text). (TIF) [file pgen.1004603.s001.tif]

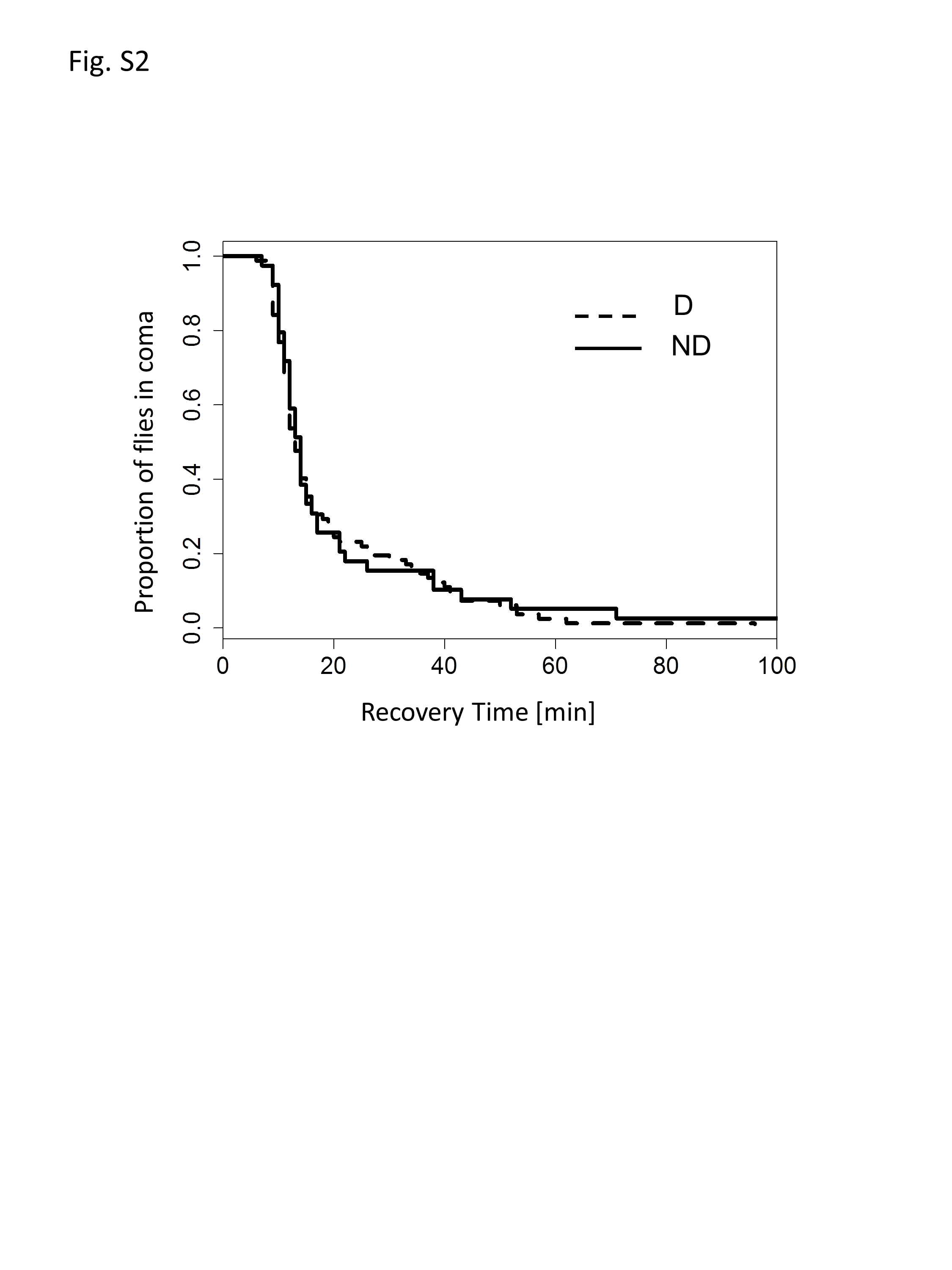

Supplement: Figure S2 — Comparison of CCRt in diapausing and non-diapausing females. Survival curves showing recovery from coma of wild-type females (Hu) following 12 days in diapause inducing conditions. Following CCRt measurement the females were dissected and the reproductive state was determined. Diapausing (n = 82) and non-diapausing (n = 39) females show similar CCRt. (TIF) [file pgen.1004603.s002.tif]

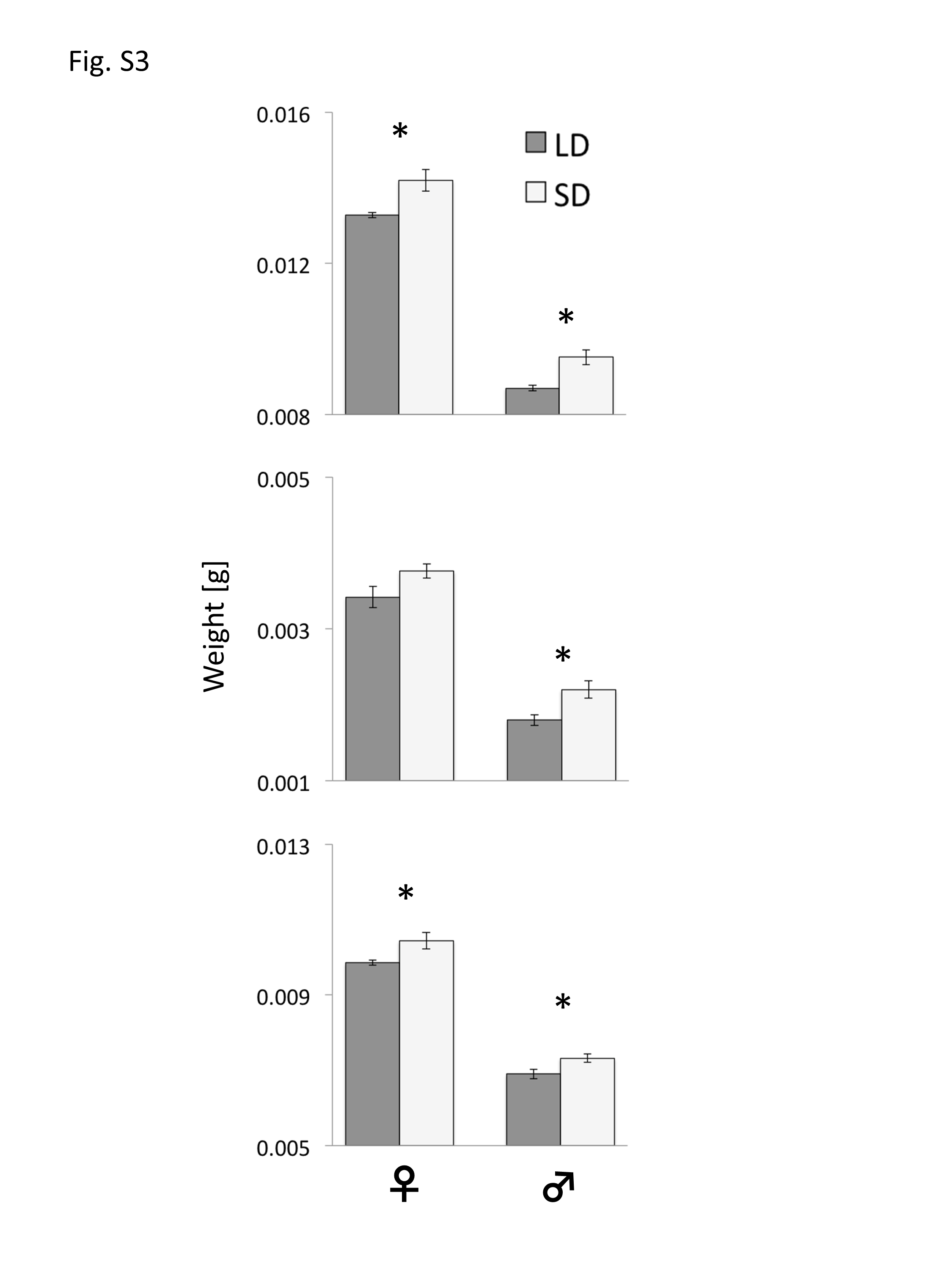

Supplement: Figure S3 — Fresh/dry weights of Drosophila under different photoperiods. The fresh weight (top), the dry weight (middle) and the water content (bottom) were measured in females (left bars) and males (right bars) raised in long (16 hr) and short (8 hr) day. Measurements are based on 4–5 replicate pools of 10 flies. The error bars represent SE. (TIF) [file pgen.1004603.s003.tif]

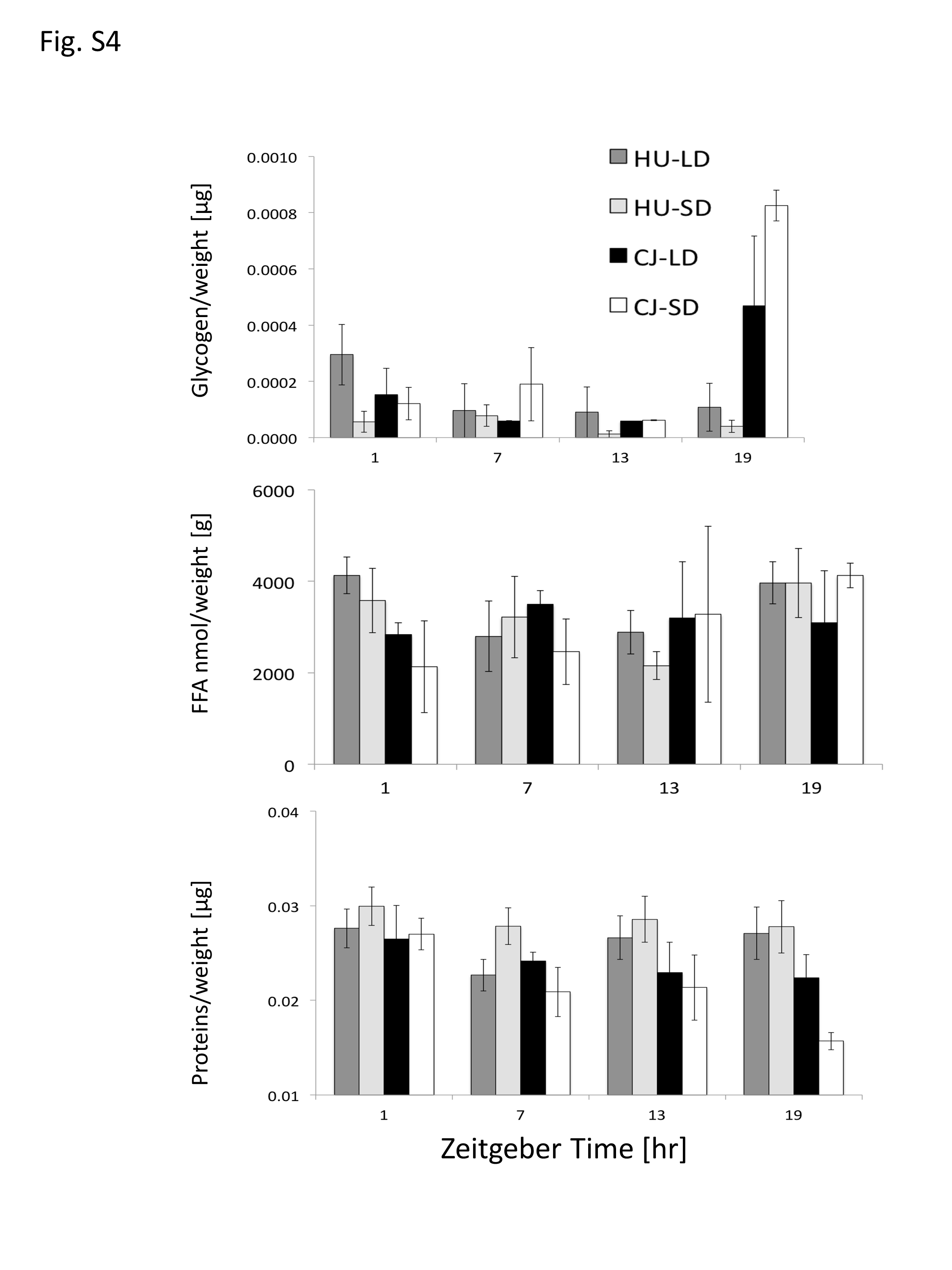

Supplement: Figure S4 — The effect of photoperiod on metabolite level. The level of glycogen (top), free fatty acids (middle) and total protein (bottom) was measured in female wild-type (HU) and mutant (ClkJRK) flies, under long and short day. Samples were collected at four different time points. Glycogen assays are based on 3–6 pools of 10 flies each. Free fatty acids assays are based on 2–3 replicates (10 flies each), and total protein 3–9 pools (10 flies each). Error bars represent SE. (TIF) [file pgen.1004603.s004.tif]

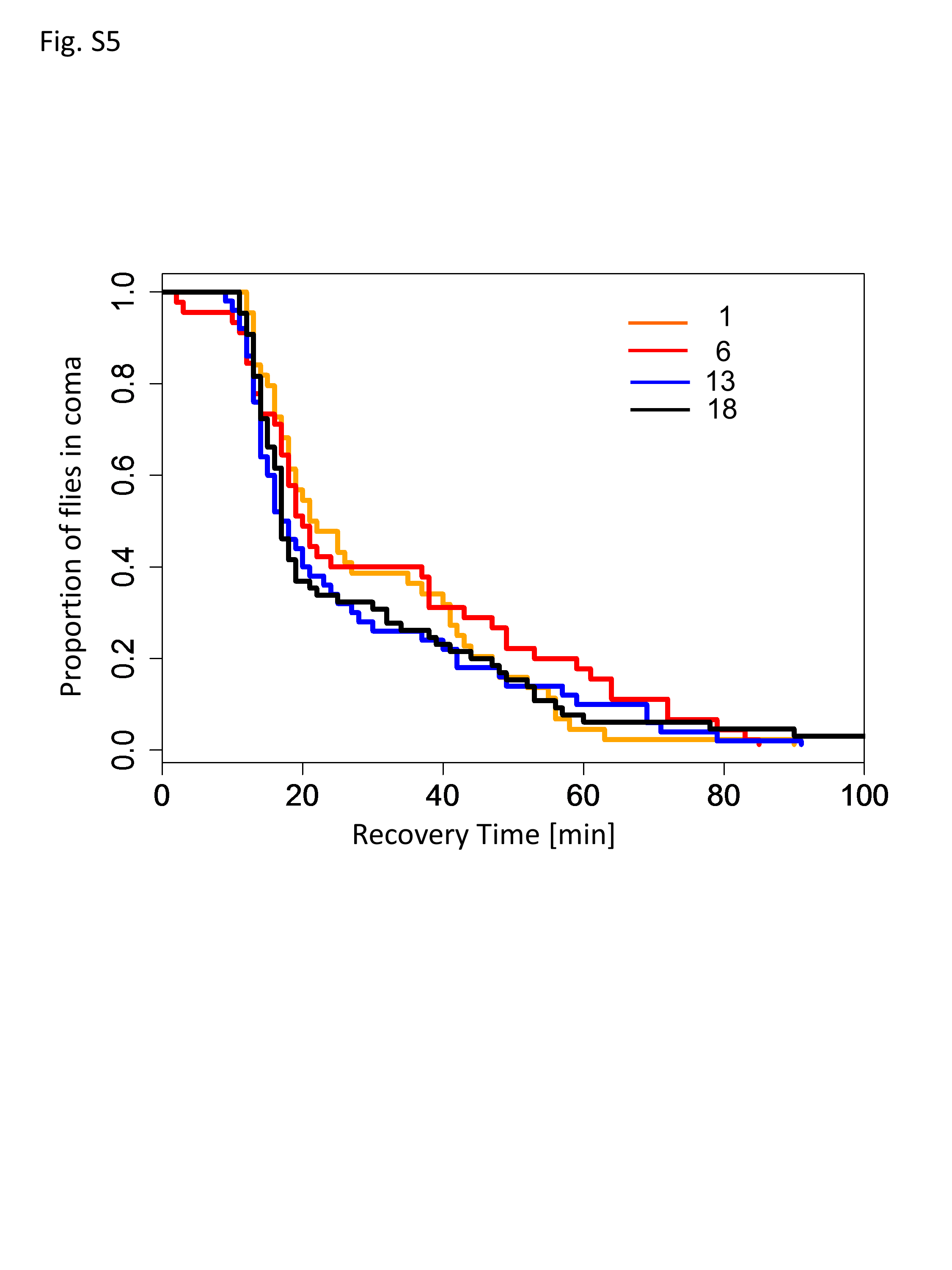

Supplement: Figure S5 — CCRt at different times of the day. Survival curves of wild-type females (Hu) showing similar recovery time from coma at different Zts. Females were developed at LD 12∶12 (n = 44–65 flies). (TIF) [file pgen.1004603.s005.tif]

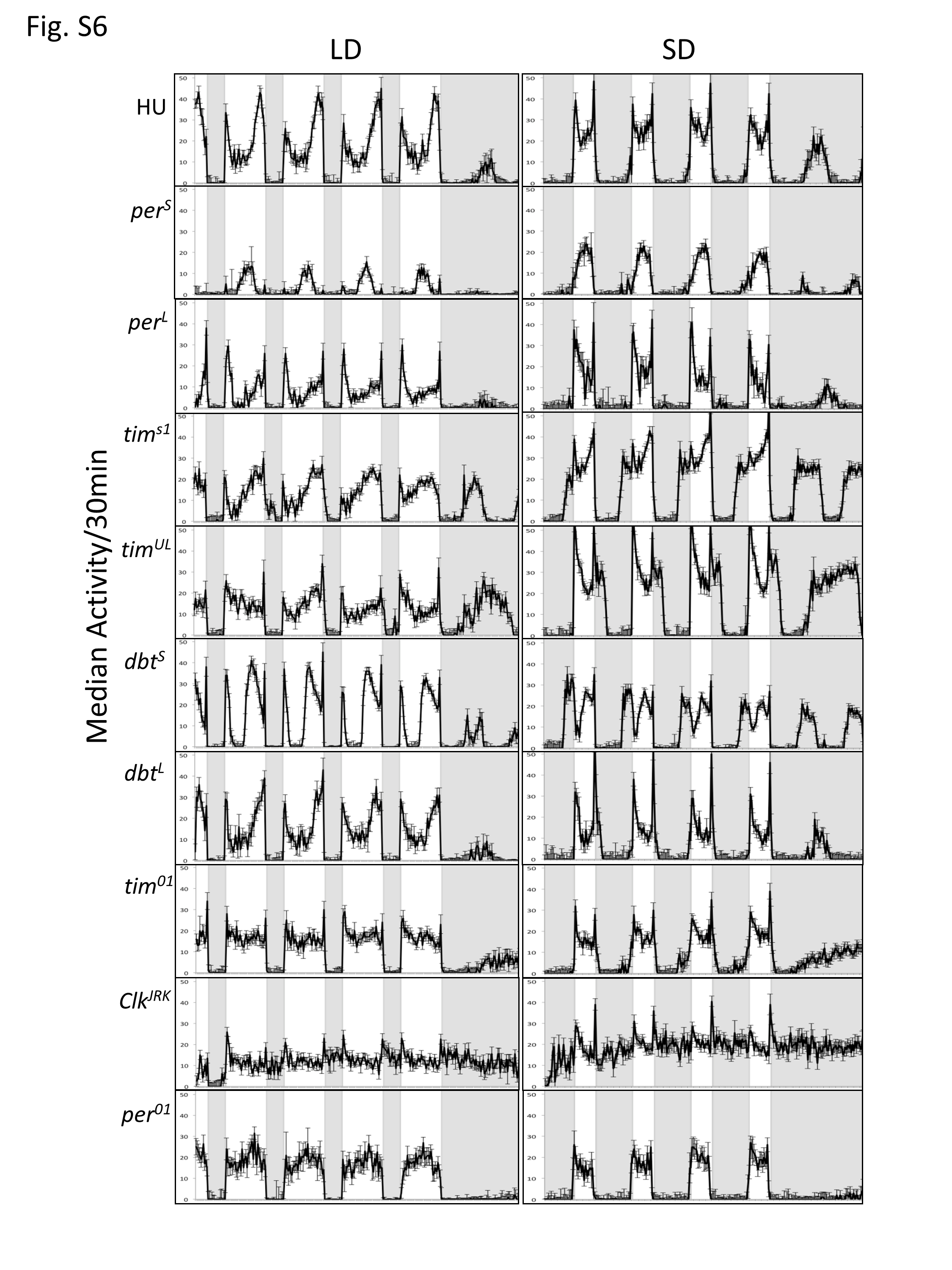

Supplement: Figure S6 — Locomotory activity profiles of clock mutants. The activity profiles of mutant female flies in long (left) and short (right) day is shown, followed by one day in DD (n = 16–35 flies). Experiment carried at 19.5°C, which was also used in the CCRt experiments. (TIF) [file pgen.1004603.s006.tif]

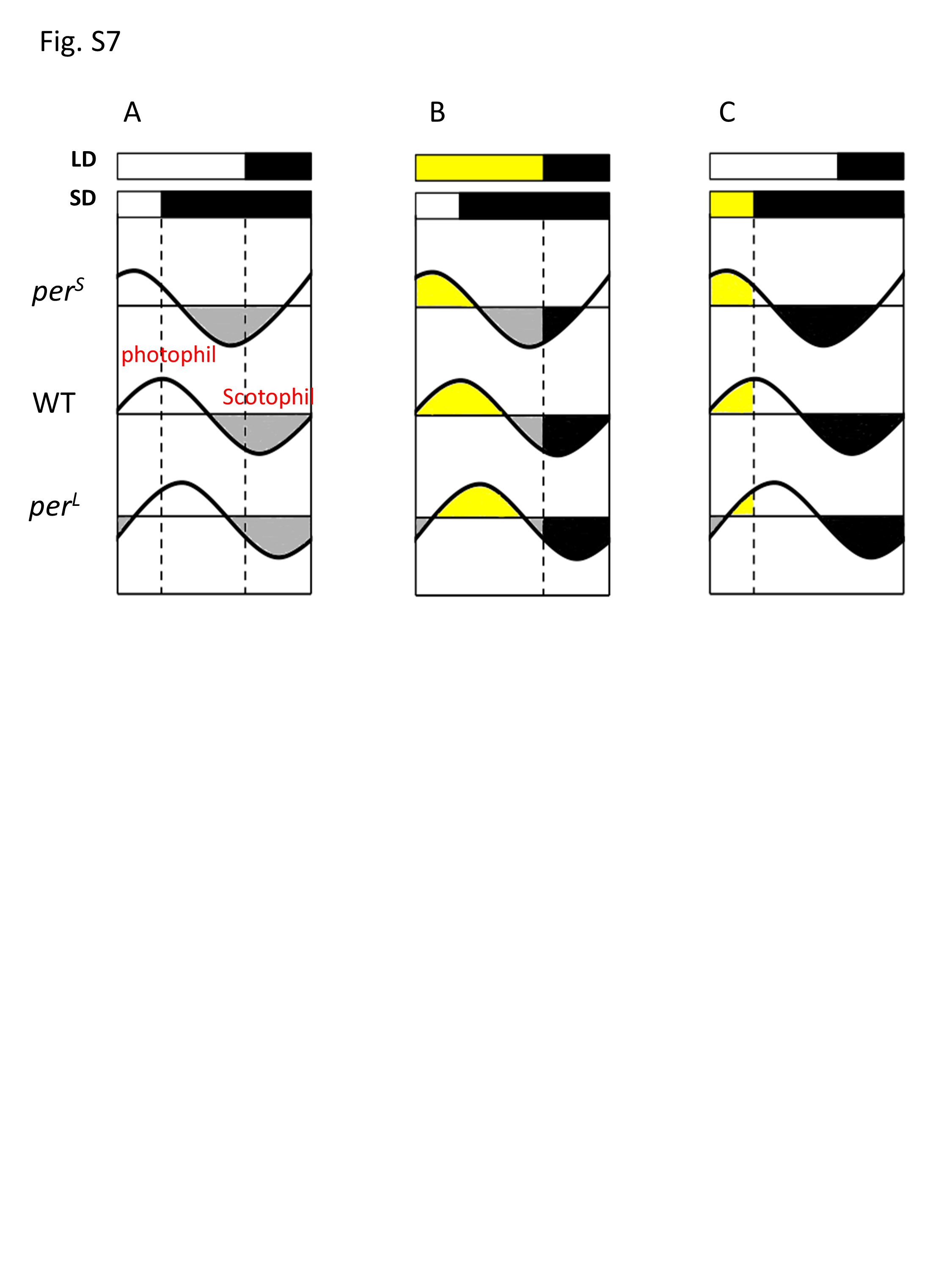

Supplement: Figure S7 — Predicted behaviour of long and short mutants under external coincidence model. A: Assuming that mutants and WT flies have the same oscillation waveform, that the waveform remains unchanged under long and short photoperiods, the scotophil and photophil phases of each strains may lie at different time of the day (perL delayed compared to perS). B: Under long days, perL mutants have a larger portion of their scotophil phase in darkness compared to per S, that leads to a short day response (better cold adapted than the other strains). This fits the hypothesis shown in Figure 3. C: Under short-days, however, perS and WT flies have the entire scotophil phase in darkness, and perS mutants have in addition the largest part of the photophil phase during the day. Therefore, perS mutants should be best winter-adapted. This was not found in the experiments. Consequently, the external coincidence model can only explain part of the results. (TIF) [file pgen.1004603.s007.tif]
